# Regulation of Tumor Suppressor p53 and HCT116 Cell Physiology by Histone Demethylase JMJD2D/KDM4D

**DOI:** 10.1371/journal.pone.0034618

**Published:** 2012-04-13

**Authors:** Tae-Dong Kim, Sangphil Oh, Sook Shin, Ralf Janknecht

**Affiliations:** University of Oklahoma Health Sciences Center, Oklahoma City, Oklahoma, United States of America; The University of Arizona, United States of America

## Abstract

JMJD2D, also known as KDM4D, is a histone demethylase that removes methyl moieties from lysine 9 on histone 3 and from lysine 26 on histone 1.4. Here, we demonstrate that JMJD2D forms a complex with the p53 tumor suppressor *in vivo* and interacts with the DNA binding domain of p53 *in vitro*. A luciferase reporter plasmid driven by the promoter of p21, a cell cycle inhibitor and prominent target gene of p53, was synergistically activated by p53 and JMJD2D, which was dependent on JMJD2D catalytic activity. Likewise, overexpression of JMJD2D induced p21 expression in U2OS osteosarcoma cells in the absence and presence of adriamycin, an agent that induces DNA damage. Furthermore, downregulation of JMJD2D inhibited cell proliferation in wild-type and even more so in p53^−/−^ HCT116 colon cancer cells, suggesting that JMJD2D is a pro-proliferative molecule. JMJD2D depletion also induced more strongly apoptosis in p53^−/−^ compared to wild-type HCT116 cells. Collectively, our results demonstrate that JMJD2D can stimulate cell proliferation and survival, suggesting that its inhibition may be helpful in the fight against cancer. Furthermore, our data imply that activation of p53 may represent a mechanism by which the pro-oncogenic functions of JMJD2D become dampened.

## Introduction

The JMJD (Jumonji C domain) family comprises ∼30 proteins in mammals that are characterized by the Jumonji C (JmjC) domain. In many, but not all JMJD proteins, this JmjC domain has catalytic activity and mediates the demethylation of histone lysine residues [1], [2]. Thus, JMJD proteins are involved in the dynamic modulation of histone posttranslational modification that governs virtually all processes within the cell, including regulation of gene transcription, epigenetic silencing, heterochromatin formation, genomic imprinting and DNA repair. Accordingly, dysregulation of JMJD protein activity can lead to diseases such as cancer [3], [4].

One subfamily of JMJD proteins consists of JMJD2A–D in humans [5]. Whereas JMJD2A–C are ∼120 kDa proteins and possess double PHD and Tudor domains, which are implicated in binding to methylated histones [6], JMJD2D is only half the size and lacks PHD and Tudor domains. In contrast to JMJD2A–C, the whole open reading frame of JMJD2D is encoded in one exon, hinting that the JMJD2D gene evolved from a reverse-transcribed JMJD2 mRNA. In addition, two other genomic sequences (JMJD2E and F) are similar to JMJD2D, but it remains unclear whether these DNA sequences are expressed into respective proteins [5]. Furthermore, whereas JMJD2A–C demethylate tri- and dimethylated histone 3 (H3) on both K9 and K36 [7]–[10], JMJD2D is unable to demethylate H3K36. However, JMJD2D demethylates not only tri- and dimethylated H3K9, but also, albeit with reduced efficiency, monomethylated H3K9 [11], [12].

Hitherto, little is known about the physiological role of JMJD2D, also called KDM4D for lysine demethylase 4D. The first function of JMJD2D revealed was in androgen signaling [13]. JMJD2D was reported to interact with the ligand binding domain of the androgen receptor and thereby activate androgen-dependent gene transcription. This ability of JMJD2D to stimulate the androgen receptor was dependent on its catalytic activity, which is consistent with the fact that especially trimethylated H3K9 is a repressive chromatin mark [14]. Moreover, JMJD2D is not an essential protein, since respective knock-out mice are viable and display no obvious phenotype. However, consistent with JMJD2D mRNA being most highly expressed in testes, H3K9 methylation in spermatids was altered in JMJD2D^−/−^ mice: trimethylated H3K9 accumulated in round spermatids and di-/monomethylated H3K9 in elongated spermatids that have no detectable trimethylated H3K9 [15], corroborating that JMJD2D is an effective *in vivo* demethylase of H3K9. Despite these changes of H3K9 methylation in spermatids, JMJD2D^−/−^ male mice surprisingly showed no decline in fertility [15].

Lysine methylation is not restricted to histones, but appears to occur on many proteins including estrogen receptor, androgen receptor, the RelA subunit of the NF-kappaB transcription factor, the coactivator PCAF and the basal transcription factor TAF10 [16]–[21]. In particular, multiple lysine methylation sites were identified in the tumor suppressor p53 that activate or repress its function depending on which lysine is modified and what the modification status is (mono- or dimethylated) [22]–[25]. While respective methyltransferases were uncovered that target p53, it remains largely unknown which enzymes are capable of demethylating p53. We wondered if JMJD2D might demethylate p53 and thus started to analyze the relationship between JMJD2D and p53. In addition, we explored herein whether JMJD2D affects the physiology of human HCT116 colon cancer cells.

## Materials and Methods

### Coimmunoprecipitation Assay

Human embryonal kidney HEK293T cells (American Type Culture Collection CRL-11268) were seeded into 6-cm dishes coated with poly-L-lysine and grown in DMEM media supplemented with 10% fetal bovine serum, 100 u/ml penicillin and 100 µg/ml streptomycin at 37°C in a humidified atmosphere containing 10% CO_2_ [26]. At 25% confluency, cells were transiently transfected by the calcium phosphate coprecipitation method [27] with 5.5 µg pBluescript KS^+^ (Stratagene), 1.5 µg empty pcDNA3 vector or pcDNA3-p53, and 2 µg empty pEV3S vector or Flag-tagged expression plasmid for JMJD2D, HSPBAP1 [11], SMCX [28], JHDM1A or JMJD6 [29]. The DNA-calcium phosphate coprecipitate was allowed to form and transfect cells for 12 h in an atmosphere containing 3% CO_2_. Then, cells were washed twice with 2 ml phosphate-buffered saline and grown thereafter for 36 h in an atmosphere containing 10% CO_2_. After that, cells were lysed in 50 mM Tris-HCl (pH 7.4), 150 mM NaCl, 50 mM NaF, 0.25 mM Na_3_VO_4_, 0.2 mM DTT, 0.5% NP-40, 2 µg/ml aprotinin, 10 µg/ml leupeptin, 1 µg/ml pepstatin A, 1 mM phenylmethylsulfonyl fluoride and immunoprecipitations performed with anti-Flag M2 (Sigma F3165) or anti-p53 DO-1 (Santa Cruz Biotechnology sc-126) monoclonal antibodies essentially as described before [30]. Similarly, endogenous JMJD2D in HCT116 cells (American Type Culture Collection CCL-247) was immunoprecipitated with anti-JMJD2D antibodies (Aviva Systems Biology ARP35946). Precipitates were then subjected to SDS polyacrylamide gel electrophoresis followed by Western blotting and coprecipitated proteins revealed utilizing M2 or DO-1 antibodies employing enhanced chemiluminescence [31].

### GST Pull-downs

Glutathione *S*-transferase (GST) fusion proteins were produced in *Escherichia coli* and purified according to standard procedures [32]. Cell extract containing Flag-tagged JMJD2D protein was prepared from transiently transfected HEK293T cells as described before [33]. This cell extract was then incubated with GST fusion proteins bound to glutathione agarose beads in 20 mM HEPES, 50 mM NaCl, 1 mM DTT, 0.01% Tween-20, 0.5 mM phenylmethylsulfonyl fluoride for 3 h at 4°C. After three washes in the same buffer [34], any Flag-tagged JMJD2D bound to GST fusion proteins was revealed by Western blotting utilizing M2 monoclonal antibodies.

### Luciferase Assay

HEK293T cells were grown in poly-L-lysine-treated 12-wells as described above. When reaching 25% confluency, cells were transiently transfected by the calcium phosphate coprecipitation method [35] with 200 ng of luciferase reporter constructs driven by the human p21 promoter (−2324/+100), the human matrix metalloproteinase-1 (MMP-1) promoter [36], or the cytomegalovirus (CMV) promoter/enhancer [37]. In addition, 50 ng empty pcDNA3 vector or pcDNA3-p53, 300 ng empty pEV3S vector or Flag-JMJD2D, and 1.8 µg pBluescript KS^+^ (Stratagene) were cotransfected. After 10 h, cells were washed twice with 1 ml phosphate-buffered saline and then incubated for another 36 h in media [38]. Thereafter, cells were washed once with 1 ml phosphate-buffered saline and lysed in 0.3 ml of 25 mM Tris-HCl (pH 7.8), 2 mM EDTA, 2 mM DTT, 1% Triton X-100 and 10% glycerol [39]. Luciferase activity was then determined in a luminometer [40].

### Chromatin Immunoprecipitation Assay

This assay was essentially performed as described before [41] with JMJD2D (Aviva Systems Biology ARP35946), p53 DO-1 (Santa Cruz Biotechnology sc-126) and H3K9me_3_ (Upstate 07–442) antibodies. As a control, the cytoplasmic Rcl protein [42] was immunoprecipitated. To amplify a p21 promoter fragment, the primer pair 5′-GTGGCTCTGATTGGCTTTCTG-3′ and 5′-CCAGCCCTGTCGCAAGGATC-3′ was employed [43] and amplified DNA resolved and visualized on an ethidium bromide-stained agarose gel [44].

**Figure 1:**
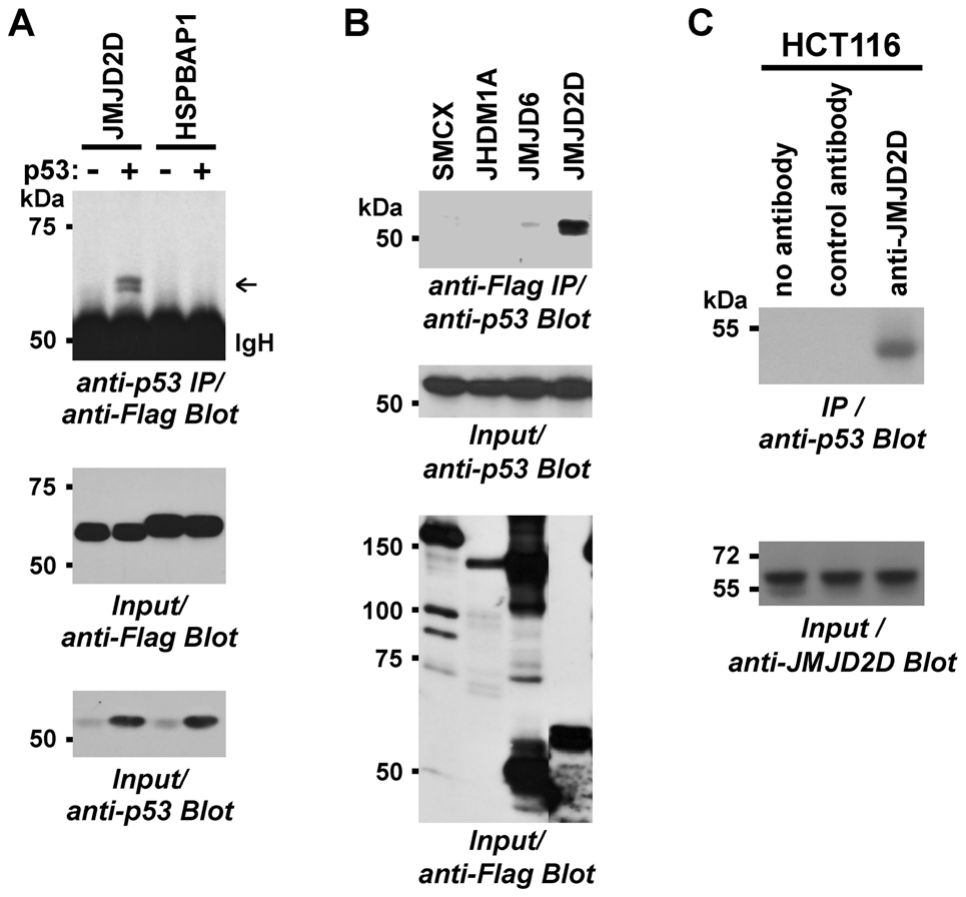
Binding of JMJD2D to p53. (**A**) Flag-tagged JMJD2D or HSPBAP1 were coexpressed with p53 in HEK293T cells. After anti-p53 immunoprecipitation (IP), coprecipitated proteins were revealed by anti-Flag immunoblotting (top panel). The bottom two panels show input levels of Flag-tagged proteins or p53. IgH, immunoglobulin heavy chain. (**B**) Indicated Flag-tagged JMJD proteins were coexpressed with p53 in HEK293T cells. After anti-Flag immunoprecipitation, coprecipitated p53 was detected by anti-p53 Western blotting (top panel). The middle and bottom panels show p53 and Flag-JMJD protein input levels, respectively. (**C**) HCT116 cell extracts were challenged with no, control or anti-JMJD2D antibodies and coprecipitated p53 detected by immunoblotting (top panel). The bottom panel shows that respective JMJD2D input levels were equal.

### Generation of Stably Transfected U2OS Cells

Flag-JMJD2D, Flag-JMJD2D-H192A or pEV3S were employed to transfect U2OS cells (American Type Culture Collection HTB-96) by the calcium phosphate coprecipitation method as described above. Two days after transfection, cells were split at a ratio of 1∶100, 1∶200, 1∶400 and 1∶800 and grown until visible colonies were formed. Twenty colonies were picked, expanded and analyzed by Western blotting for the expression of Flag-tagged proteins. One cell line each for the transfected plasmids was then selected for further analysis. These stably transfected U2OS cells were treated with DMSO or 1 µM adriamycin (stock solution of 4 mM dissolved in DMSO) for 24 h, cell extracts prepared by boiling in Laemmli buffer [45] and analyzed by Western blotting [46] employing antibodies against JMJD2D (Aviva Systems Biology ARP35946), p21 (Santa Cruz Biotechnology sc-756), actin (Sigma A2066), H3K9me_3_ (Upstate 07–442), H3K9me_2_ (Upstate 07–441), H3K9me_1_ (Upstate 07–450) or histone H3 (Santa Cruz Biotechnology sc-10809).

### RT-PCR

Stably transfected U2OS cells were treated with DMSO or 1 µM adriamycin (stock solution of 4 mM dissolved in DMSO) for 18 h and RNA then prepared utilizing Trizol (Invitrogen) according to the manufacturer's instructions. RT-PCR was performed employing the Access RT-PCR kit (Promega) with p21-RT-for-2 (5′-GTTTCTGCGGCAGGCGCCATG-3′) and p21-RT-rev-2 (5′-GGGCGGATTAGGGCTTCC-3′) primers under the following conditions: 48°C for 45 min, 95°C for 2 min, 20 or 25 cycles of 94°C for 30 s, 58°C for 30 s, 72°C for 30 s, and a final extension step at 72°C for 2 min. DNA was electrophoresed on an agarose gel and visualized by ethidium bromide staining. The expected length of the p21 cDNA was 520 bp. A 226 bp control GAPDH (glyceraldehyde 3-phosphate dehydrogenase) cDNA was amplified as described previously [47].

### Retroviral Infection

The retroviral expression vector pSIREN-RetroQ (Clontech) was employed to express shRNAs. The following sequences within human JMJD2D were targeted: GCCAGAGAGACCTATGATA (#1) and ACCACGTTTGCTTGGCATA (#3). To generate respective retrovirus, HEK293T cells were cotransfected with the shRNA vector and necessary packaging plasmids [48]. Supernatants containing retrovirus were collected 48 h and 72 h after transfection, passed through a 0.45 µm filter, and concentrated by precipitation with poly(ethylene glycol)-8000. Then, cells were infected three times (every 12 h) with retrovirus [49]. Subsequently, cells were selected for two days in 1 µg/ml puromycin, as the pSIREN-RetroQ vector encodes the respective antibiotica resistance gene. Then, cells were treated without or with 1 µM adriamycin for indicated times and JMJD2D and actin protein expression determined as described above.

### Cell Proliferation Assay

50,000 retrovirally infected cells were plated into 6-cm dishes. Cells were then treated with 1 µM adriamycin or DMSO as a control for 72 h, harvested, stained with trypan blue and live cells totaled with the help of a Countess cell counter (Invitrogen) according to the manufacturer's recommendations. Experiments were performed in triplicate and averages with standard error are shown. Statistical significance was determined with an unpaired, two-tailed *t*-test.

### Flow Cytometry

Retrovirally infected cells were grown in 6-cm dishes for 72 h. Then, cells were harvested, washed in phosphate-buffered saline, fixed in 63% ethanol for 12 h at 4°C, and incubated with 40 µg/ml propidium iodide and 50 µg/ml RNase A for 30 min at 37°C [50]. After that, the proportion of sub-G1 cells was determined by sampling 20,000 events with a FACSCalibur flow cytometer utilizing Cell Quest software (BD Biosciences). Triplicate experiments were performed and statistical significance determined with an unpaired, two-tailed *t*-test.

**Figure 2:**
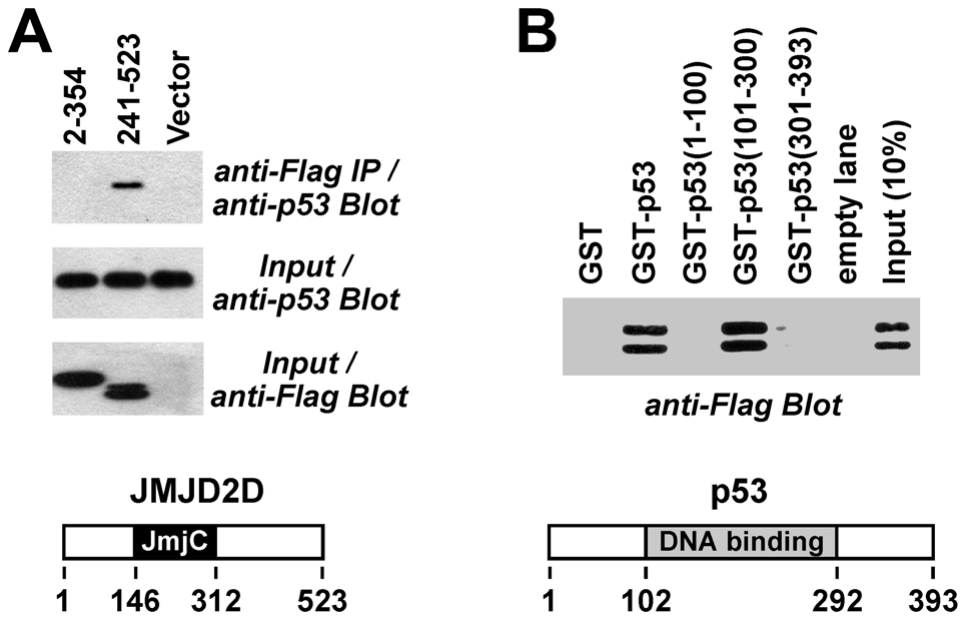
Mapping of interaction domains. (**A**) Indicated Flag-tagged amino acids of JMJD2D were coexpressed with p53 in HEK293T cells and complex formation assessed in coimmunoprecipitation assays as in Fig. 1B. A sketch of human JMJD2D highlighting its JmjC domain is presented at the bottom. (**B**) Comparable amounts of GST or indicated GST-p53 fusion proteins were bound to glutathione agarose. After incubation with Flag-tagged JMJD2D, bound JMJD2D was revealed by anti-Flag Western blotting. The location of the DNA binding domain within p53 is indicated in the sketch at the bottom.

## Results

### Interaction between JMJD2D and p53

Previously, it was reported that the human tumor suppressor p53 can be mono-/dimethylated on K370, K372 and K382, yet only one protein, lysine-specific demethylase 1, has been identified that is able to demethylate p53 by targeting K370 [22]–[25]. We speculated that JMJD2D might demethylate p53 and thus analyzed the ability of JMJD2D to demethylate p53 peptides encompassing di- or monomethylated K370, K372 or K382 *in vitro*. However, we found that JMJD2D was unable to demethylate any of these peptides (not shown). At the same time, we reasoned that a potential demethylase might interact with its substrate and therefore analyzed in parallel whether JMJD2D would form a complex with p53 *in vivo*. To this end, we coexpressed Flag-tagged JMJD2D and p53 in HEK293T cells, immunoprecipitated with anti-p53 antibodies and then probed for the presence of JMJD2D by anti-Flag Western blotting. Indeed, JMJD2D was revealed when p53 was coexpressed (Fig. 1A, top panel). In contrast, another JMJD protein, HSPBAP1, did not coimmunoprecipitate with p53 (Fig. 1A, top panel), although comparable levels of Flag-tagged HSPBAP1 and JMJD2D were expressed (Fig. 1A, middle panel), indicating that our immunoprecipitation assay was specific. To confirm this complex formation between p53 and JMJD2D, we performed also reverse-order coimmunoprecipitation assays. Again, we observed that p53 and JMJD2D formed a complex *in vivo* and once more this was specific, since three other JMJD proteins (SMCX, JHDM1A, JMJD6) did not coimmunoprecipitate p53 (Fig. 1B). Finally, we assessed whether endogenous p53 and JMJD2D also form complexes. For this, we employed human HCT116 colon cancer cells that endogenously express both p53 and JMJD2D. Immunoprecipitation with anti-JMJD2D antibodies, but not with control or no antibodies, pulled down p53 (Fig. 1C) demonstrating that endogenous JMJD2D and p53 interact *in vivo*.

**Figure 3:**
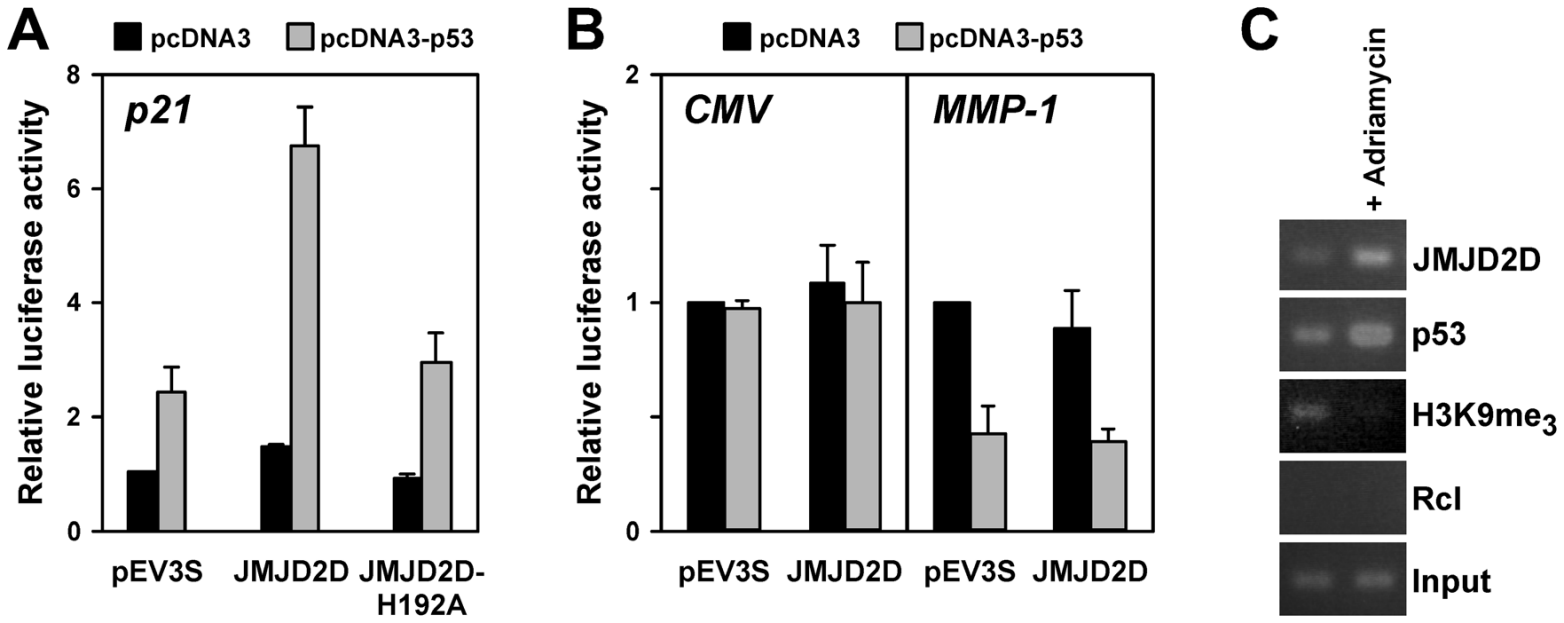
Activation of the p21 promoter by JMJD2D in HEK293T cells. (**A**) Activity of a p21 luciferase reporter construct upon cotransfection of control vector pEV3S or wild-type or H192A JMJD2D is depicted. As indicated, empty vector pcDNA3 or pcDNA3-p53 was also transfected. (**B**) Analogous, response of a CMV or MMP-1 luciferase reporter construct to cotransfection of p53 and/or JMJD2D. (**C**) Chromatin immunoprecipitation assay in HCT116 cells treated without and with adriamycin.

Next, we analyzed which protein domains might be responsible for the JMJD2D-p53 interaction. In coimmunoprecipitation experiments, the N-terminal 354 amino acids of JMJD2D were unable to pull down p53, but the C-terminal amino acids 241–523 did (Fig. 2A). Thus, the catalytic JmjC domain stretching from amino acid 146–312 does not mediate this interaction. This provides an explanation why SMCX, JHDM1A or JMJD6 and possibly many more JMJD proteins do not interact with p53, because their common denominator, the JmjC domain, is not sufficient to facilitate this interaction. Conversely, we employed GST pull-down assays to interrogate which amino acids of p53 are capable of binding to JMJD2D. As shown in Fig. 2B, full-length p53 as well as its central DNA binding domain interacted *in vitro* with JMJD2D, whereas the N- and C-terminus of p53 did not.

**Figure 4:**
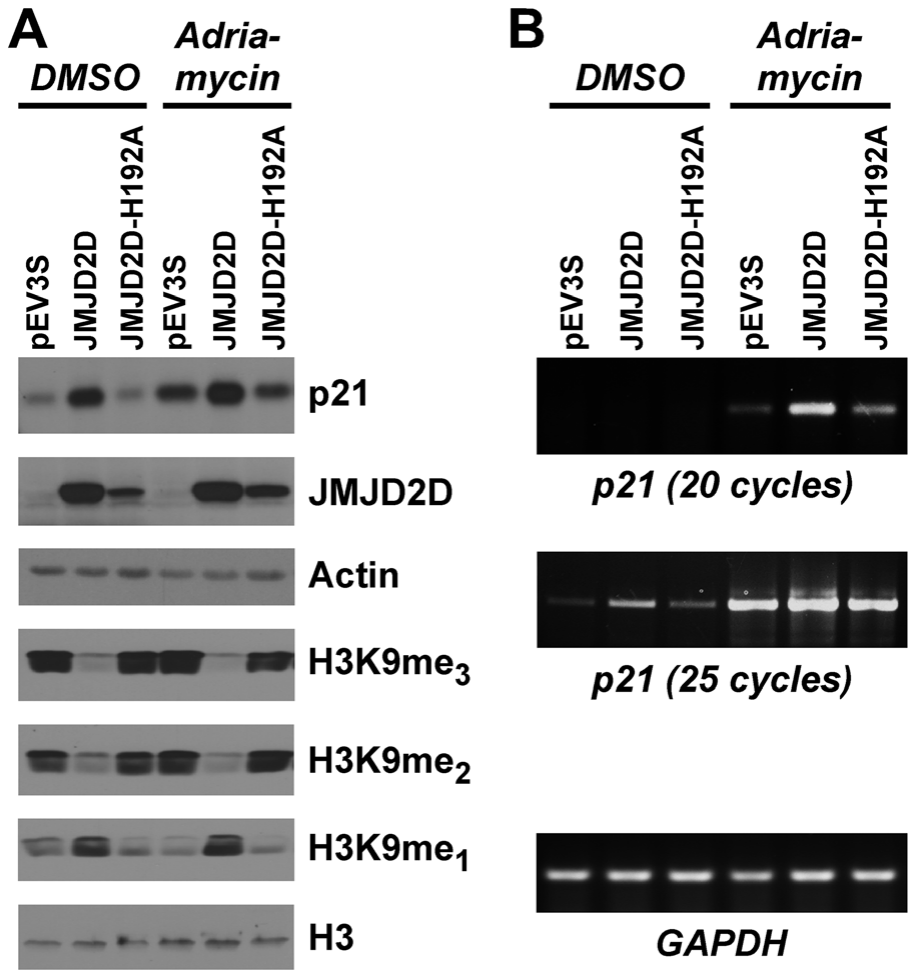
Stimulation of p21 expression by JMJD2D in U2OS cells. (**A**) Wild-type or H192A JMJD2D or empty vector pEV3S were stably transfected into U2OS cells. Expression of indicated proteins was assessed by Western blotting in cells treated for 24 h with 1 µM adriamycin or DMSO as a control. (**B**) RNA was isolated from stably transfected U2OS cells and RT-PCR analyses were performed. Shown is the amplification of p21 cDNA and, as a control, GAPDH cDNA.

### Stimulation of p53 by JMJD2D

The physical interaction with p53 suggested that JMJD2D modulates the transcriptional activity of p53. One prominent p53 target gene is the p21 cell cycle inhibitor [51]. Thus, we employed a p21 luciferase reporter gene to analyze the impact of JMJD2D on p53-dependent transcription. As expected, expression of p53 alone resulted in increased p21 promoter activity in HEK293T cells (Fig. 3A). Expression of JMJD2D slightly enhanced luciferase activity, whereas the combination of p53 and JMJD2D displayed a synergistic activation of the p21 promoter. Notably, mutating the catalytic center of JMJD2D by replacing the crucial histidine residue 192 with alanine abrogated this synergy (see H192A mutant in Fig. 3A). Furthermore, JMJD2D's ability to coactivate was specific, since the CMV promoter was unaffected by p53 and/or JMJD2D overexpression and the MMP-1 promoter was repressed by p53, whereas JMJD2D had no effect (Fig. 3B). We conclude that JMJD2D is capable of stimulating p53-dependent transcription and that its catalytic activity is required to do so.

**Figure 5:**
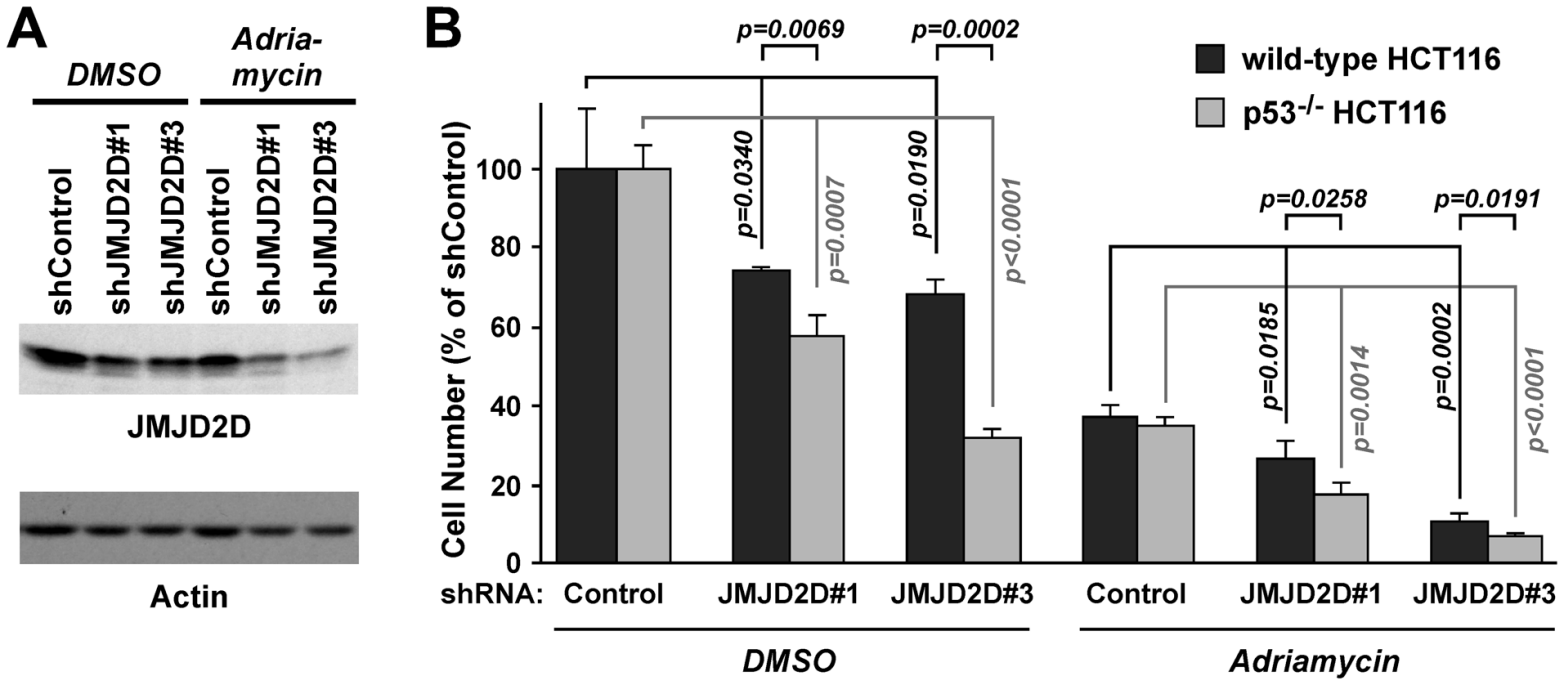
JMJD2D depletion results in reduced cell proliferation. (**A**) HCT116 cells expressing control or JMJD2D shRNA were treated with 1 µM adriamycin or with DMSO as a control for 24 h. Downregulation of JMJD2D was assessed by Western blotting; immunoblotting for actin served as a loading control. (**B**) Wild-type or p53^−/−^ HCT116 cells were challenged with control or JMJD2D shRNA (#1 or #3) and then treated without or with 1 µM adriamycin for 72 h. The number of cells were counted and presented as percent of the control shRNA for each wild-type and p53^−/−^ HCT116 cells. Statistical significance of differences between various experimental conditions is indicated in the graph.

**Figure 6:**
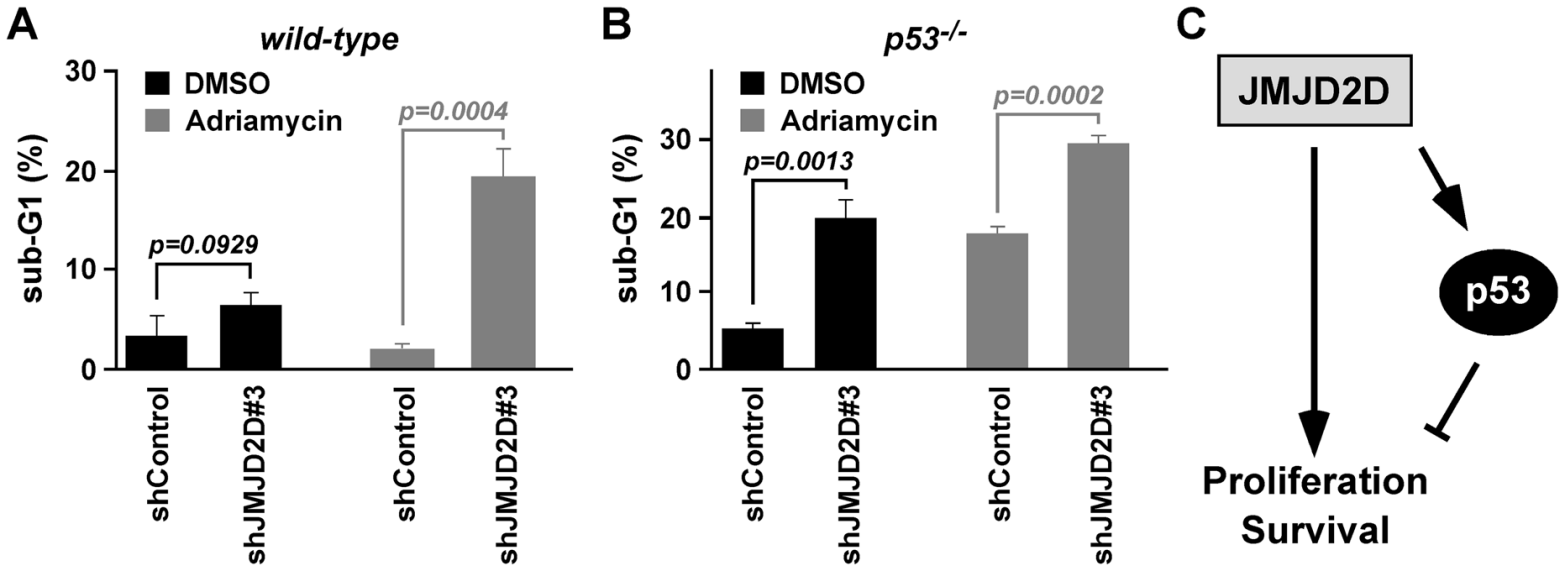
JMJD2D protects from apoptosis. (**A**) The level of sub-G1 HCT116 cells was determined in cells that expressed control shRNA or JMJD2D shRNA#3. Cells were treated for 72 h with 1 µM adriamycin or DMSO as indicated. (**B**) The same in case of p53^−/−^ HCT116 cells. (**C**) Model of JMJD2D action.

To further confirm this notion, we studied binding of JMJD2D to the endogenous p21 promoter in HCT116 cells by chromatin immunoprecipitation assays. Moreover, we treated these cells with the DNA damaging agent adriamycin, which is known to induce p53 activity and hence p21 expression. As expected, adriamycin treatment enhanced binding of p53 to the p21 promoter (Fig. 3C). Likewise, JMJD2D binding increased upon adriamycin treatment, and simultaneously H3K9 trimethylation became reduced. These data suggest that JMJD2D binds to the p21 gene promoter together with p53 and possibly contributes to p21 gene activation by reducing levels of trimethylated H3K9.

Next, we established human U2OS osteosarcoma cells that stably express either wild-type JMJD2D or its catalytic H192A mutant. Whereas we did not detect any significant amounts of endogenous JMJD2D in wild-type U2OS cells, strong levels of JMJD2D were observed in stably transfected U2OS cells (Fig. 4A). Moreover, both in control and in adriamycin-treated cells, JMJD2D expression led to enhanced p21 protein levels (Fig. 4A), further substantiating that JMJD2D is a coactivator of p53. In addition, similar as reported before for HEK293T cells [11], we observed that JMJD2D overexpression in U2OS cells led to globally reduced levels of tri- and dimethylated H3K9, whereas levels of monomethylated H3K9 were enhanced. In contrast, the H192A mutant did not change global H3K9 methylation levels and also did not raise p21 proteins levels, again suggesting that catalytic activity of JMJD2D is required for its ability to coactivate p53 (Fig. 4A). However, there is the caveat that we were unable to establish cells expressing JMJD2D-H192A at the same high level as wild-type JMJD2D; this may suggest that the catalytic mutant exerts toxic effects in U2OS cells.

To demonstrate that JMJD2D indeed affects transcription of the endogenous p21 gene, we also isolated RNA from our stably transfected U2OS cells. RT-PCR experiments were then performed, which showed that p21 mRNA levels were strongly enhanced by JMJD2D, whereas JMJD2D-H192A overexpression had very little effect (Fig. 4B). This was observed both in the presence and absence of adriamycin. Altogether, our data indicate that JMJD2D stimulates the transcription of the p53 target gene, p21.

### Impact of JMJD2D on Cell Proliferation and Survival

Next, we attempted to study the physiological role of JMJD2D and chose human HCT116 colon cancer cells for analysis, because matching wild-type and p53^−/−^ HCT116 cells are available [52] and because endogenous JMJD2D was readily detectable. First, we generated two shRNAs targeting JMJD2D, both of which significantly reduced JMJD2D levels in HCT116 cells (Fig. 5A). Then we assessed how downregulation of JMJD2D would affect proliferation. In comparison to HCT116 cells infected with a retrovirus expressing a control shRNA, virus delivering JMJD2D#1 or JMJD2D#3 shRNA caused a significant decrease in cell number (Fig. 5B). Please note that different cell numbers do not necessarily equate with different rates of proliferation, since this may (partly) be due to altered apoptosis levels. However, we only observed a ∼3% increase in apoptotic cells when downregulating JMJD2D (see below and Fig. 6A), which is unlikely to account for the ∼30% decrease of cell number in JMJD2D-depleted cells; thus, we believe that the main cause of decreased cell number upon JMJD2D depletion is reduced cell proliferation. Moreover, treatment of cells with adriamycin resulted expectedly in a reduction of cell number and JMJD2D depletion further reduced cell numbers. Next, we assessed the same in p53^−/−^ HCT116 cells. Again, we observed that JMJD2D depletion led to reduced cell numbers both in the absence and presence of adriamycin; interestingly, it appears that JMJD2D depletion had a greater effect in p53^−/−^ compared to wild-type HCT116 cells (Fig. 5B).

In addition, we assayed the impact of JMJD2D depletion on cell death, which was monitored by determining cells that had a sub-G1 DNA content. In wild-type HCT116 cells, JMJD2D knock-down resulted in a ∼2-fold increase of apoptotic cells (3.3% to 6.5%; Fig. 6A), whereas apoptosis was more pronouncedly enhanced upon JMJD2D depletion in p53^−/−^ HCT116 cells (5.5% to 20.0%; Fig. 6B). Similarly, JMJD2D knock-down resulted in more apoptosis in adriamycin-treated p53^−/−^ compared to wild-type HCT116 cells (29.3% versus 19.5%; Fig. 6A and 6B). Collectively, these data indicate that JMJD2D promotes cell survival in both p53^+/+^ and p53^−/−^ HCT116 cells.

## Discussion

Here, we have provided evidence that the histone demethylase JMJD2D is a coactivator of p53. JMJD2D can interact with the DNA binding domain of p53 and synergize with p53 to induce expression of p21, a cell cycle inhibitor and prominent p53 target gene [51]. Thus, it is likely that JMJD2D will cooperate with p53 at many more of its target gene promoters, but comprehensive genome-wide studies are needed to corroborate this experimentally. One mechanism by which JMJD2D may stimulate p53-dependent transcription is by especially demethylating trimethylated H3K9, a mark of repressed genes [14], and accordingly we observed that catalytic activity of JMJD2D was required for its ability to stimulate the p21 gene promoter. In addition to targeting H3K9, JMJD2D was recently shown to demethylate H1.4K26 [12], [53]. Moreover, histone H1.4 is enriched in heterochromatin, confers transcriptional repression and recruits heterochromatin binding protein 1 when methylated [54]–[56]. Thus, JMJD2D may also induce p53 target genes by removing repressive methylation marks from H1.4K26. Interestingly, JMJD2A has recently been shown to act as a p53 corepressor [43], indicating that JMJD2D is distinct from JMJD2A and can perform an opposite function in transcriptional regulation.

Downregulation of JMJD2D in HCT116 colon cancer cells revealed that JMJD2D is a pro-proliferative molecule, showing for the first time a physiological role for this demethylase. This is, at first sight, counterintuitive because of JMJD2D's function as a p53 coactivator, as this tumor suppressor normally reduces cell proliferation, in part by upregulating the p21 cell cycle inhibitor [57]. However, JMJD2D may primarily stimulate cell proliferation through to-be-deciphered pathways, whereas JMJD2D's ability to coactivate p53 dampens this pro-proliferative function (Fig. 6C). If so, one would predict that JMJD2D has a greater impact on cell proliferation in p53-negative cells, and indeed we observed that JMJD2D depletion in p53^−/−^ HCT116 cells resulted in a greater decrease of cell proliferation compared to wild-type HCT116 cells.

In addition, JMJD2D depletion led to more sub-G1 cells, which is indicative of apoptosis. This is suggestive of JMJD2D being a pro-survival molecule. Again, this is somewhat counterintuitive with JMJD2D being a p53 coactivator, since p53 upregulates pro-apoptotic genes such as Puma [51]. But again, one can invoke that JMJD2D primarily stimulates cell survival via pathways not involving p53, whereas its action through p53 may stifle this pro-survival activity. If so, JMJD2D's effect on survival should be greater in the absence of p53. And in fact, downregulation of JMJD2D resulted in more sub-G1 cells in p53^−/−^ HCT116 cells compared to corresponding wild-type cells.

The ability of JMJD2D to stimulate cell proliferation and survival would be consistent with a pro-oncogenic role of JMJD2D. In this regard, JMJD2D resembles JMJD2A that likewise appears be required for maximal HCT116 cell growth [43]. Notably, several reports suggest that JMJD2 genes might be overexpressed in various cancers. For instance, JMJD2C gene amplification has been observed in esophageal squamous cell carcinomas, lung sarcomatoid carcinoma, medulloblastomas and breast tumors [58]–[62]. Further, JMJD2B or JMJD2C mRNA are overexpressed in medulloblastomas, JMJD2A-C mRNA upregulation was noted in prostate tumors and JMJD2B/C mRNA levels are also enhanced in many breast tumors [8], [61]–[63]. More importantly, JMJD2B or JMJD2C overexpression induced transformed phenotypes in non-cancerous breast cells, whereas their downregulation in breast cancer cells inhibited cell proliferation *in vitro* or tumorigenesis in xenograft mouse models [62]–[65]. Collectively, these data suggest that JMJD2 proteins might be proto-oncoproteins, yet it remains to be studied whether this holds true for JMJD2D. If so, JMJD2D might be a valid target for cancer therapy like other JMJD proteins [66]. Interestingly, compounds related to 2-oxoglutarate, which is a cofactor of JMJD demethylases, and succinate, which is generated from 2-oxoglutarate as a by-product, have been shown to inhibit JMJD2D catalytic activity [67], [68] and could therefore form a starting point in the development of small molecule drugs.

In conclusion, our study provides evidence that JMJD2D may modulate the transcriptional activity of p53, which is one of the most important tumor suppressors and found mutated in half of all human tumors [69]. In addition, we provide evidence that JMJD2D is a pro-proliferative and pro-survival molecule, which suggests that small molecules targeting JMJD2D could be beneficial in the treatment of cancer.
